# Single versus multiple firesetting: an examination of demographic, behavioural and psychological factors

**DOI:** 10.1080/13218719.2023.2272904

**Published:** 2024-01-11

**Authors:** Katie Sambrooks, Nichola Tyler, Theresa A. Gannon

**Affiliations:** a CORE-FP, School of Psychology, University of Kent, Canterbury, UK; b Kent and Medway NHS and Social Care Partnership Trust, Maidstone, UK; c School of Psychology, Keele University, Keele, UK; d School of Psychology, Victoria University of Wellington, Wellington, New Zealand

**Keywords:** arson, assessment, dynamic risk factors, firesetting, repeat firesetting, treatment, treatment needs

## Abstract

Deliberate firesetting is a prevalent issue. While a number of psychological treatment needs have been identified for adults who set fires, their association with multiple firesetting has received limited attention. This study examined whether demographics, offence histories, firesetting behaviours and psychometric assessments of psychological vulnerabilities hypothesised to be associated with firesetting discriminate between adults who have set only one fire and those who have set multiple fires. Participants (N =128) were recruited from prisons and categorised according to whether they self-reported having set only a single fire (n = 60) or multiple fires (n = 68) as an adult. Our findings provide evidence that identification with fire, antisocial attitudes and anger-related cognition and arousal are associated with multiple firesetting, and therefore represent key treatment targets for interventions. Furthermore, a history of setting fires within prison was the largest unique predictor of multiple firesetting (odds ratio, OR = 6.83), highlighting the urgent need for research on institutional firesetting.

Deliberate firesetting refers to all acts of intentionally starting a fire, including, but not limited to, the legal offence of arson (Gannon & Pina, 2010). This behaviour is both highly problematic and prevalent across many countries (Tyler, Gannon, et al., 2019). For example, 63,723 deliberate fires were attended by the Fire and Rescue Service in England over the last 12 months (Home Office, 2022b), which resulted in 58 deaths and 883 injuries (Home Office, 2022c). In the United States, 540 fatalities and 1320 injuries were attributed to deliberate fires over a 12-month period (Campbell, 2021). Consequently, deliberate firesetting is now considered to be an international public health concern (Tyler, Gannon, et al., 2019). Adult-perpetrated deliberate firesetting is a particularly serious issue. In England and Wales, adults represented 86.2% of criminal proceedings for criminal damage and arson in the year ending June 2022 (Ministry of Justice, 2022). Similarly, in the United States, adults accounted for 74% of the arrests for arson over the 10 years prior to 2021 (Federal Bureau of Investigation, 2022). Deliberate firesetting by adults also appears to be a persistent behaviour. A recent meta-analysis established that approximately one in five adults with a history of firesetting, who had not received firesetting-specific treatment, engaged in further firesetting behaviour (Sambrooks et al., 2021). Given that adult-perpetrated deliberate firesetting is a behaviour that is likely to be recurrent, it is of critical importance that evidence-based assessments and treatment programmes are available to reduce the risk of repeat firesetting among adults.

One approach to tackling repeat offending is to align treatment efforts with the principles of the risk–need–responsivity (RNR) model (Andrews & Bonta, 2010; Bonta & Andrews, 2016). According to the RNR model, for an intervention to be effective it must specifically target an individual’s criminogenic needs. These criminogenic needs represent dynamic risk factors that are modifiable and associated with reductions in reoffending when adequately treated (Andrews & Bonta, 2010; Bonta & Andrews, 2016). Therefore, advancing knowledge of dynamic risk factors associated with repeat firesetting is essential. The multi-trajectory theory of adult firesetting (M-TTAF; Gannon et al., 2012; Gannon et al., 2022) represents the most up-to-date and comprehensive theory of adult-perpetrated deliberate firesetting, and therefore complements the RNR model by suggesting potential dynamic risk factors for firesetting. Specifically, the M-TTAF describes four clusters of psychological vulnerabilities hypothesised to be associated with deliberate firesetting: (a) inappropriate fire interest and/or inappropriate fire scripts; (b) offence supportive attitudes (firesetting specific and/or generally criminal); (c) self and emotional regulation problems; and (d) communication issues.

Previous research has provided evidence that the four clusters of psychological vulnerability within the M-TTAF distinguish adults who have set fires from individuals with no firesetting history, supporting the presence of unique criminogenic needs associated with firesetting. For example, Gannon et al. (2013) demonstrated that, relative to matched comparison males who had engaged in non-fire criminal activity, imprisoned adults who had set fires held several distinct fire-specific treatment needs (i.e. significantly greater levels of identification with fire, attitudes legitimising firesetting as normal, interest in serious fire-related situations and significantly lower fire safety awareness). Gannon et al. (2013) also showed that the firesetting group demonstrated a number of unique self and emotional regulation issues, including increased experiences of anger to perceived provocation, anger-related cognition and anger arousal. Finally, with regard to communication deficits, or more broadly defined social competence issues, studies with psychiatric samples have found lower social skills and greater social isolation among patients with a history of firesetting than non-firesetting patients (Hagenauw et al., 2015; Wilpert et al., 2017).

## Dynamic risk factors for multiple firesetting

While identification of characteristics that distinguish firesetting and non-firesetting adults has been critical in highlighting the need for specialist assessment and treatment protocols, these studies do not demonstrate whether issues in these areas are associated with repeat firesetting. Therefore, the extent to which these factors represent dynamic risk factors and the potential importance of these for risk assessments cannot be determined from the research detailed thus far. There has been a paucity of theoretically informed research, with the majority of studies focusing on descriptive comparisons of single-fire and multiple-fire individuals (Doley et al., 2011). While true risk factors are best identified through longitudinal research (Bonta & Andrews, 2016), cross-sectional studies that compare single and multiple fire individuals provide evidence of factors that are associated with repeat firesetting, and thus offer a useful starting point for identifying factors for inclusion in firesetting assessment and treatment protocols.

The majority of cross-sectional studies have focused on static or historical factors. For example, individuals who have set multiple fires are more likely than individuals who have only set a single fire to have been a victim of physical or sexual abuse (Bell et al., 2018), hold a history of childhood firesetting (Rice & Harris, 1996; Tyler et al., 2015) and have a previous diagnosis of a personality disorder (Dickens et al., 2009; Ducat et al., 2015; Rice & Harris, 1991; Wyatt et al., 2019) or Axis 1 disorder (Ducat et al., 2015). In addition, multiple-fire individuals have been found to have more previous arson convictions (Ducat et al., 2015; Edwards & Grace, 2014; Rice & Harris, 1996; Sapsford et al., 1978; Tyler et al., 2015) and more charges or convictions for any offence type (Ducat et al., 2015; Field, 2016) than single-fire individuals. While these studies have identified potential static risk factors that can be useful for informing risk assessments of the likelihood of repeat firesetting, their clinical utility is limited as they yield little information regarding potentially modifiable variables that should be targeted in treatment.

There has been scant focus on examining dynamic risk factors for repeat firesetting. Nevertheless, one of the most consistent findings in the limited literature relates to the association between inappropriate fire interest and repeat firesetting. Several studies have found that both adults and youths who have set multiple fires demonstrate more interest in fires than individuals who have only set one fire (Dickens et al., 2009; Kennedy et al., 2006; MacKay et al., 2006; Rice & Harris, 1991). For example, Tyler et al. (2015) found forensic patients had 15 times greater odds of having set multiple fires if they were recorded as having an inappropriate interest in fire or explosives in their clinical notes. However, when using a psychometric measure of inappropriate fire interest (the Four Factor Fire Scales; FFFS) with imprisoned male adults, Ó Ciardha, Barnoux, et al. (2015) found that they were unable to discriminate between single-fire (*n* = 74) and multiple-fire (*n* = 41) individuals using the Serious Fire Interest subscale. In contrast, the Identification with Fire subscale did accurately discriminate between the two groups, providing initial evidence of another potential dynamic risk factor for multiple firesetting.

Due to the lack of theoretically informed investigations of repeat firesetting, the remaining psychological vulnerabilities (as hypothesised by the M-TTAF) have received limited attention. A small number of studies have indirectly examined emotion regulation issues. For example, Rice and Harris (1991) found high security patients who had a history of setting multiple fires were less likely to have a history of interpersonal aggression than patients who had only set one fire, according to their clinical records. In addition, Wyatt et al. (2019) found that multiple-fire individuals were more often recorded as having an external locus of control and as demonstrating impulsivity than single-fire individuals.

While these studies indicate that individuals who have set multiple fires may differ in terms of their criminogenic needs relative to individuals who have set only one fire, the findings have often been drawn from psychiatric data coded retrospectively (Doley et al., 2011; Tyler et al., 2015). This is problematic because the method of initial assessment of the risk factor is typically unclear, and the subjectivity of this assessment and the subsequent coding are frequently unknown. Current best practice when assessing the psychological vulnerabilities of adults who have set fires is to administer psychometric measures (Gannon et al., 2022). Consequently, further research is needed to establish whether these proposed differences in criminogenic needs are demonstrated through the psychometric measures that are commonly administered in firesetting assessments and used to guide treatment. It is hoped such research will inform future assessment protocols, as well as provide further direction when treatment planning for adults who have set fires.

## Research questions and hypotheses

This study compares the characteristics of adults who self-reported having set only one fire with adults who self-reported having set multiple fires during adulthood. This study aims to address five research questions. First, we investigate whether there are any differences between adults who have set only one fire and adults who have set multiple fires in terms of their background factors (i.e. demographics, offence histories). Second, we examine whether the firesetting behaviour (e.g. context of firesetting) of adults who have set only one fire differs from that of adults who have set multiple fires. These analyses will inform which variables are later examined as covariates of firesetting psychological vulnerabilities. Next, we investigate whether scores from psychometric assessments of psychological vulnerabilities are correlated with the self-reported number of fires set during adulthood. We hypothesise that number of fires set will be positively correlated with scores on the Four Factor Fire Scales (FFFS; Ó Ciardha, Tyler, et al., 2015). In addition, we examine whether adults who have only set one fire score differently on assessments of psychological vulnerabilities than adults who have set multiple fires. We hypothesise that adults who have set multiple fires will scores higher on the FFFS than adults who have set only one fire. Finally, we examine to what extent assessments of the psychological vulnerabilities can predict whether an adult has set multiple fires.

## Method

### Participants

Participants were originally recruited from several UK prison establishments as part of two wider treatment studies examining individuals with a history of firesetting. Participants with poor English literacy and those experiencing active psychosis or suicidal ideation, or at risk of hostage taking were excluded from the original studies. A total of 128 participants from the two studies were included in the current research. Seventy-three participants were initially recruited as part of the original evaluation of the Firesetting Intervention Programme for Prisoners (FIPP; Gannon, 2017) by Gannon et al. (2015). Fifty-five participants were recruited from across three prisons as part of a new, ongoing FIPP evaluation, as described by Sambrooks and Tyler (2019). All participants were male and had a recorded history of deliberate firesetting or fire-related risk behaviours (e.g. attempted firesetting or repeated threats to set fires) during adulthood (i.e. post the age of 18 years). While a conviction for firesetting was not necessary, the participants’ firesetting behaviour was determined to meet the inclusion criteria for firesetting treatment (see Gannon, 2017). Participants had not undertaken any firesetting-specific treatment at the time of measure completion, but they may have previously completed other general offending behaviour programmes in prison. The mean age of the combined samples was 33.61 years (*SD* = 11.42). Sentence length ranged from 2 to 432 months, with participants serving an average sentence of 79.03 months (*SD* = 68.86, *n* = 114) for an average of 2.22 index offences^1^ (*SD* = 1.96, *n* = 114).

Participants were categorised into two groups based on the number of deliberate fires they self-reported having set in adulthood:^2^ single-fire individuals (*n* = 60) and multiple-fire individuals (*n* = 68). The number of self-reported fires was used as opposed to the number of arson convictions as deliberate firesetting is an offence where officially recorded figures tend to underestimate the prevalence of the behaviour (Gannon et al., 2022). The number of self-reported fires ranged from 1 to 1000. The median number of fires set during adulthood by multiple-fire individuals was 4 (interquartile range, IQR = 2, 10).

### Measures

#### Background factors

Background factors spanned demographic variables (e.g. age, ethnicity), psychiatric variables (e.g. mental health diagnosis) and offence history. These variables were obtained from file reviews and clinical interviews with participants. Offence history was collected from Police National Computer (PNC) records in participants’ prison files.

#### Firesetting behaviour variables

A number of self-report variables relating to participants’ past firesetting behaviour were collected via clinical interviews. This included the number of fires set in childhood (i.e. below the age of 18 years old), their age at their first childhood firesetting incident and their age at their last (most recent) firesetting incident. Several dichotomous (yes/no) variables, primarily relating to the context of their firesetting, were also obtained: whether they deny any firesetting incident they have been accused of, whether they had ever set a cell fire,^3^ whether they had engaged in any self-directed firesetting (e.g. using fire as a form of self-harm or in a suicide attempt), whether they had engaged in any face-to-face violence via firesetting,^4^ and whether they had engaged in any indirect violence via firesetting.^5^

#### Psychological vulnerabilities

Self-report psychometric measures assessing elements of each of the four areas of psychological vulnerability in the M-TTAF were administered by trained researchers and clinicians. Measure selection was dependent on which cohort participants were recruited from. Measures were presented to participants in a randomised order.

##### Fire-related measures

The Four Factor Fire Scales (FFFS; Ó Ciardha, Tyler et al., 2015) combines items from three fire-related measures: the Fire Interest Rating Scale (Murphy & Clare, 1996), the Fire Attitude Scale (Muckley, 1997) and the Identification with Fire Questionnaire (Gannon et al., 2011). The Fire Interest Rating Scale examines an individual’s fascination with or attraction to fire and consists of 14 items describing fire-related situations (e.g. ‘Watching a house burn down’). Participants are asked to rate how interested they would be in each of the situations on a scale of 1 (*upsetting/frightening)* to 7 (*exciting, fun* *or lovely*). The Fire Attitude Scale consists of 19 items and examines an individual’s attitudes towards fire. Participants respond to items such as ‘Setting just a small fire can make you feel a lot better’ on a scale from 1 (*strongly disagree*) to 5 (*strongly agree*). The Identification with Fire Questionnaire consists of 17 items and assesses the extent to which an individual relates to or identifies with fire (e.g. ‘Fire is almost part of my personality’). Participants also respond to this measure on a 5-point scale (1 = *strongly disagree* to 5 = *strongly agree*).

In the Four Factor Fire Scales, these measures are combined to form four subscales that have been empirically determined via factor analysis (see Ó Ciardha, Tyler, et al., 2015). These four subscales examine (a) identification with fire (e.g. ‘Fire is almost part of my personality’; 11 items), (b) serious fire interest (e.g. ‘Striking a match to set fire to a building’; 7 items), (c) perceived fire safety awareness (e.g. ‘I know a lot about how to prevent fires’; 6 items), and (d) firesetting as normal (e.g. ‘Most people have set a few small fires just for fun’; 7 items). The total score on the Four Factor Fire Scales reflects an individual’s overall fire interest, attitudes and affiliation to fire, and perceived fire safety awareness (Ó Ciardha, Tyler, et al., 2015). Gannon et al. (2013) have reported questionable to good psychometric properties for the subscales when administered with imprisoned males with a history of firesetting (identification with fire, α = .88; serious fire interest, α = .86; perceived fire safety awareness, α = .68; normalisation of firesetting, α = .73) and excellent reliability for the total score (α = .90).^6^ This measure was completed by both cohorts of participants.

##### Offence-supportive attitude measures

The Measure of Criminal Attitudes and Associates Part B (MCAA–Part B; Mills & Kroner, 1999) is a 46-item measure of antisocial attitudes. It consists of four subscales that examine the extent to which the individual holds attitudes that endorse (a) violence (e.g. ‘It’s understandable to hit someone who insults you’; 12 items), (b) sentiments of entitlement (e.g. ‘Taking what is owed you is not really stealing’; 12 items), (c) antisocial intent (e.g. ‘I could see myself lying to the police’; 12 items), and (d) criminal associates (e.g. ‘I always feel welcome around criminal friends’; 10 items). Participants are asked to either agree or disagree with each item. The psychometric properties of the MCAA–Part B are well established with forensic populations (see Gannon et al., 2013; Mills et al., 2002, 2004). This measure was completed by both cohorts of participants.

##### Self and emotional regulation measures

The Novaco Anger Scale and Provocation Inventory (NAS–PI; Novaco, 2003) are two related measures. The NAS (60 items) examines anger experiences across four subscales: cognition (e.g. ‘Once something makes me angry, I keep thinking about it’), arousal (e.g. ‘When I get angry, I stay angry for hours’), behaviour (e.g. ‘My temper is quick and hot’) and anger regulation (e.g. ‘If I feel myself getting angry, I can calm myself down’). Participants are asked to select one of three response options (1 = *never*, 2 = *sometimes,* or 3 = *always true*). The NAS Total Score is based on the Cognitive, Arousal and Behaviour subscales. Due to only having access to subscale scores and not item-level data from Gannon et al. (2015), this Total Score has been calculated as the average of the *t* scores for each of the mentioned subscales. The Provocation Inventory (PI; 25 items) provides an index of an individual’s anger intensity across a range of potentially provocative situations (e.g. ‘Someone else gets credit for work that you did’), using a 4-point scale (1 = *not at all angry* to 4 = *very angry*). The NAS–PI has well-established psychometric properties for forensic and non-forensic samples (see Culhane & Morera, 2010; Gannon et al., 2013; Novaco, 2003). These measures were completed by both cohorts of participants.

The Nowicki–Strickland Locus of Control (Nowicki, 1976) is a 40-item measure of an individual’s perception of whether events are internally or externally controlled (e.g. ‘Are some people just born lucky?’). Participants respond with either a yes or a no answer. Acceptable psychometric properties of the scale have been established with forensic (Gannon et al., 2013) and non-forensic samples (Nowicki & Duke, 1974). This measure was completed only by the Gannon et al. (2015) cohort of participants.

The Barratt Impulsiveness Scale (BIS; Patton et al., 1995) is a 30-item measure designed to tap into three sub-traits of impulsiveness: (a) Attentional Impulsiveness, which involves making quick decisions (e.g. ‘I am a careful thinker’), (b) Motor Impulsiveness, which involves acting without thinking (e.g. ‘I act on the spur of the moment’) and (c) Non-Planning Impulsiveness, which involves a lack of forethought (e.g. ‘I am more interested in the present than the future’). Participants were asked to respond on a 4-point scale (1 = *rarely/never* to 4 = *almost always/always*). Evidence for these factors was found in samples of undergraduates, psychiatric inpatients and adult male prisoners (Patton et al., 1995). In the current study, this measure demonstrated acceptable psychometric properties (Attentional Impulsiveness, α = .71; Motor Impulsiveness, α = .66; Non-Planning Impulsiveness, α = .68). This measure was only completed by the Sambrooks and Tyler (2019) cohort.

The Coping Strategies Inventory–Short Form (CSI–SF; Addison et al., 2007) is a 16-item measure assessing the presence of four coping strategies. The items form four subscales: (a) Problem–Focused Engagement (e.g. ‘I make a plan of action and follow it’, (b) Problem-Focused Disengagement (e.g. ‘I hope the problem will take care of itself’), (c) Emotion-Focused Engagement (e.g. ‘I let my feelings out to reduce the stress’) and (d) Emotion-Focused Disengagement (e.g. ‘I keep my thoughts and feelings to myself’). Engagement and Disengagement scores are also calculated. Participants respond on a 5-point scale (1 = *never* to 5 = *very often*). Addison et al. (2007) found the CSI–SF to have acceptable levels of internal consistency with non-forensic populations. In the current study, alphas ranged from .51 to .76. This measure was only completed by the Sambrooks and Tyler (2019) cohort.

##### Social competence measures

The Revised University of California, Los Angeles (UCLA) Loneliness Scale (Russell et al., 1980) is a 20-item measure of emotional loneliness (e.g. ‘There is no one I can turn to’), rated on a 4-point scale (1 = *never* to 4 = *often*). Good psychometric properties have been established, including with imprisoned males (*α* = .86; Gannon et al., 2013). This measure was completed only by the Gannon et al. (2015) cohort of participants.

The Simple Rathus Assertiveness Schedule–Short Form (Jenerette & Dixon, 2010) is a 19-item measure of assertiveness across a variety of social situations (e.g. ‘I am quick to say what I think’) rated on a 6-point scale (1 = *very much unlike me* to 6 = *very much like me*). Jenerette and Dixon (2010) reported good internal reliability (α = .81), which was also evidenced in the Gannon et al. (2013) study with males with a history of firesetting (α = .81). This measure was completed only by the Gannon et al. (2015) cohort of participants.

The Culture-Free Self-Esteem Inventory (Battle, 1992) is a 40-item forced-choice (yes/no) measure of self-esteem. The measure consists of three subscales that assess (a) general self-esteem (e.g. ‘Are you happy most of the time?’), (b) personal self-esteem (e.g. ‘Do you feel that you are as important as most people?’) and (c) social self-esteem (e.g. ‘Do you have many friends?’). The psychometric properties of this measure are well established (e.g. Battle, 1997), with Gannon et al. (2013) demonstrating good internal consistency with imprisoned males with a history of firesetting (Kuder-Richardson 20 (KR20) = .86). This measure was completed only by the Gannon et al. (2015) cohort of participants.

The Attachment Style Questionnaire (ASQ; Feeney et al., 1994) is a 40-item measure that assesses an individual’s attachment style in regard to general (rather than specifically romantic) relationships. Participants are asked to respond on a 6-point scale from 1 (‘totally disagree’) to 6 (‘totally agree’). Items are grouped into five subscales: (a) Confidence in self and others (e.g. ‘I am confident that other people will like and respect me’); (b) Discomfort with closeness (e.g. ‘I prefer to depend on myself rather than other people’); (c) Relationships as secondary (e.g. ‘Achieving things is more important than building relationships’); (d) Need for approval (e.g. ‘It’s important to me to avoid doing things that others won’t like’); and (e) Preoccupation with relationships (e.g. ‘I worry a lot about my relationships’). Feeney et al. (1994) report that the five scales showed adequate internal consistency when administered to university students (*α* ranging from .76 to .84). Similar figures were found in the current study (Confidence in Self and Others, α = .60; Discomfort with closeness, α = .75; Relationships as secondary, α = .64; Need for approval, α = .70; Preoccupation with relationships, α = .71). This measure was only completed by the Sambrooks and Tyler (2019) cohort.

### Procedure

All psychometric measures were administered face to face in individual sessions with participants. For the Gannon et al. (2015) cohort, participants were given the option of completing the measures themselves or having them read aloud to them by the researcher. Forty-eight of these participants selected to have them read aloud (for nine participants this information was not recorded). In the Sambrooks and Tyler (2019) cohort, all participants had the measures read aloud to them to ensure maximum comprehension.

### Analysis plan

All analyses were pre-registered with the Open Science Framework (https://osf.io/7b8qe/?view_only=d718ba59026b46b2a115ea097bf94147) and completed in RStudio. First, to identify potential covariates to be entered in later analyses, differences between the groups on background factors (relating to demographics, psychiatric history and offence history) and firesetting behaviour variables were examined using χ^2^ or *t* tests. Alternatively, Fisher’s Exact Tests were used where more than 20% of expected cell counts were less than 5, and Mann–Whitney *U* tests were used where the data were not normally distributed. Second, correlations between scores on the psychometric measures and the number of fires participants self-reported having set in adulthood were calculated. Differences between single-fire and multiple-fire individuals in terms of their scores on the psychometric measures were assessed using *t* or Mann–Whitney *U* tests. Sensitivity power analyses for these research questions were completed in GPower and are reported in Table 1.

**Table 1.** Sensitivity power analyses.

Research question	Test	Participant cohort	Effect size^a^
Are there any differences between adults who have set only one fire and adults who have set multiple fires in terms of their background factors?	χ^2^	Both	0.25
	*t*		0.50
	Mann–Whitney *U*		.51
Does the firesetting behaviour of adults who have set only one fire and adults who have set multiple fires differ?	χ^2^	Both	0.25
	*t*		0.50
	Mann–Whitney *U*		.51
Are psychometric assessments of the psychological vulnerabilities proposed by the M-TTAF correlated with number of fires set?	correlation	Both	.24
		Gannon et al. (2015)	.32
		Sambrooks and Tyler (2019)	.37
Do adults who have only set one fire score differently on psychometric assessments of the psychological vulnerabilities proposed by the M-TTAF than adults who have set multiple fires?	*t*	Both	0.50
		Gannon et al. (2015)	0.69
		Sambrooks and Tyler (2019)	0.88
	Mann–Whitney *U*	Both	0.51
		Gannon et al. (2015)	0.71
		Sambrooks and Tyler (2019)	0.90

Note: M-TTAF = multi-trajectory theory of adult firesetting.

^a^Effect size able to be detected at 80% power.

Variables that reached statistical significance (*p* < .05) and/or demonstrated a medium effect size (i.e. *d* ≥ 0.5, *r* ≥ .3, or Φ ≥ 0.3) were selected for entry into a logistic regression to assess the ability of these factors to categorise individuals on the basis of multiple firesetting, while controlling for potential covariates (demographic factors, offence history variables or firesetting behaviour variables). We did not correct for error regarding the number of univariate tests undertaken to ensure all potential variables were considered for model inclusion. The number of selected predictor variables was based on guidance by Vittinghoff and McCulloch (2007) who suggest that problems are uncommon if there are five or more outcome events per predictor variable (EPV). Therefore, no more than 12 predictor variables were selected. A receiver operating characteristic (ROC) curve was plotted to examine how well the model differentiated single-fire and multiple-fire individuals. This ROC analysis produced an area under the curve (AUC) figure, which is interpreted in line with Rice and Harris (2005) guidelines (.56 = small effect size; .64 = medium effect size; .71 = large effect size).

## Results

### Background factors

As can be seen in Table 2, the groups did not significantly differ on any demographic factor including age (*U* = 1898.50, *p* = .501, *r* = −.06), sentence length (*U* = 1680.50, *p* = .880, *r* = −.01), presence of a psychiatric diagnosis (*p* = .437, Fisher’s exact test), or history of engaging in treatment programmes whilst in prison, χ^2^(1, *N* = 90) = 0.43, *p* = .513, Φ = 0.06. There was no significant association between multiple firesetting and a dichotomised ethnicity variable (white, non-white), χ^2^(1, *N* = 128) = 0.29, *p* = .529, Φ = 0.05.

**Table 2.** Demographic and psychiatric history variables for single-fire and multiple-fire individuals.

Variable	Single fire (*n* = 60)				Multiple fires (*n* = 68)				Test statistic	95% CI	*p*	Effect size
	*M*	*SD*	% yes	*n*	*M*	*SD*	% yes	*n*				
Age	33.40	12.36		60	33.79	10.62		68	1898.50^a^	[−5.00, 3.00]	.501	−.06^c^
Sentence length (months)	77.24	60.09		58	80.86	77.25		57	1680.50^a^	[−15.00, 16.00]	.880	−.01^c^
Ethnicity									0.29^b^	—	.592	.05^e^
White			85.00	51			89.71	61				
Non-White			15.00	9			10.29	7				
Diagnosed with a mental health disorder			43.33	26			79.41	54	^f^	[0.35, 12.00]	.437	2.06^d^
Engaged in a prison-based treatment programme			66.67	40			73.53	50	0.43^b^	—	.513	.06^e^

Note: CI = confidence interval.

^a^Assumption of normality violated: Mann–Whitney *U* test. ^b^Chi-square test.

^c^Effect size measure: *r*. ^d^Effect size measure: odds ratio. ^e^Effect size measure: phi.

^f^Assumption of expected cell count violated: Fishers Exact Probability Test used.

Differences between the two groups were also investigated in terms of their offence histories as recorded by the PNC, which can be seen in Table 3. Participants who had only set one fire had significantly fewer convictions overall (*U* = 1271.50, *p* = .002, *r* = −.27) and convicted offences recorded (*U* = 1192.50, *p* = .001, *r* = −.31) than participants who had set multiple fires. When broken down by offence type, single-fire individuals had significantly fewer theft and kindred offences^7^ (*U* = 1141.00, *p* < .001, *r* = −.33), offences relating to police, courts and prison (*U* = 1425.00, *p* = .027, *r* = −.20), firearms offences (*U* = 1401.00, *p* = .011, *r* = −.20) and miscellaneous offences (*U* = 1403.00, *p =* .016, *r* = −.22). Single-fire individuals also had significantly fewer cautions (*U* = 1264.00, *p* = .003, *r* = −.27) and fewer cautionable offences (*U* = 1226.00, *p* = .001, *r* = −.29). They had also been convicted for significantly less violent non-sexual offences (*U* = 1177.00, *p* = .038, *r* = −.20).

**Table 3.** Offence histories as recorded on the PNC.

Variable	Single fire (*n* = 60)			Multiple fires (*n* = 68)			Test statistic	95% CI	*p*	Effect size
	*M*	*SD*	*n*	*M*	*SD*	*n*				
Number of index offences	2.30	2.14	56	2.14	1.79	58	1663.00^a^	[−0.00, −0.00]	.816	−.02
Number of convictions	12.16	12.24	57	18.95	15.09	65	1271.50^a^	[−10.00, −2.00]	.002**	−.27
Total number of convicted offences	28.02	38.16	57	44.17	35.74	65	1192.50^a^	[−26.00, −6.00]	.001**	−.31
Number of offences against the person	1.96	2.63	57	3.34	4.28	65	2556.50^a^	[−1.00, 0.00]	.119	−.14
Number of sexual offences	0.11	0.41	57	0.05	0.28	64	1895.00^a^	[−0.00, 0.00]	.330	−.09
Number of offences against property	2.88	3.69	57	5.51	5.35	65	1190.50^a^	[−3.00, 1.00]	<.001***	−.31
Number of fraud and kindred offences	0.89	1.90	57	1.14	6.98	63	2005.00^a^	[−0.00, 0.00]	.120	−.15
Number of theft and kindred offences	9.12	16.28	57	18.65	18.16	65	1141.00^a^	[−13.00, −2.00]	< .001***	−.33
Number of offences against the state	0	0	57	0	0	64	—	—	—	—
Number of public order offences	0.95	1.36	57	1.66	2.58	65	1693.00^a^	[−0.00, 0.00]	.377	−.08
Number of offences relating to Police, Courts and Prison	4.39	5.72	57	6.52	7.81	65	1425.00^a^	[−3.00, −0.00]	.027*	−.20
Number of drug/alcohol offences	1.18	2.11	57	1.18	1.82	65	1655.50^a^	[−0.00, 0.00]	.267	−.10
Number of offences relating to immigration	0	0	57	0	0	64	—	—	—	—
Number of firearms offences	0.74	1.49	57	0.95	1.05	65	1401.00^a^	[−1.00, −0.00]	.011*	−.23
Number of miscellaneous offences	4.96	15.00	57	4.89	7.49	65	1403.00^a^	[−2.00, −0.00]	.016*	−.22
Number of cautions	1.04	1.24	57	2.02	3.17	64	1264.00^a^	[−1.00, −0.00]	.003**	−.27
Number of cautionable offences	1.05	1.26	57	2.05	2.07	64	1226.00^a^	[−1.00, −0.00]	.001**	−.29
Total number of violent non-sexual offences	4.26	4.78	50	6.44	6.96	61	1177.00^a^	[−3.00, −0.00]	.038*	−.20
Total number of violent sexual offences	0.12	0.46	40	0.09	0.39	56	1144.00^a^	[−0.00, 0.00]	.677	−.04
Total number of firesetting offences	1.13	0.74	52	2.05	2.85	61	1471.50^a^	[−1.00, 0.00]	.482	−.07

Note: PNC = Police National Computer; CI = confidence interval.

^a^Assumption of normality violated: Mann–Whitney *U* test.

*p <.05. **p < .01. ***p < .001.

### Firesetting behaviour

As can be seen in Table 4, participants who had set multiple fires in adulthood set significantly more fires in childhood than participants who had only set one fire in adulthood (*U* = 1168.00, *p* < .001, *r* = −.31). The difference between the groups in terms of the age at which they set their first fire in childhood, *t*(61) = −1.57, *p* = .122, Cohen’s *d =* −0.41, did not reach statistical significance, although the effect size was moderate. There was no significant difference in the age at which they set their last fire (*U* = 1538.50, *p* = .910, *r* = −.01). There was no significant association between the proportion of participants who denied a firesetting incident and multiple or single firesetting, χ^2^(1, *N* = 117) = 0.33, *p* = .567, Φ = 0.05. However, a significantly greater proportion of multiple-fire individuals had set a cell fire, χ^2^(1, *N* = 128) = 17.03, *p* < .001, Φ = 0.36. There were no significant associations between multiple or single firesetting and the proportion of individuals who had engaged in self-directed firesetting, χ^2^(1, *N* = 125) = 1.36, *p* = .243, Φ = 0.10, face-to-face violence via firesetting, χ^2^(1, *N* = 123) = 0.02, *p* = .876, Φ = 0.07 or indirect violence via firesetting, χ^2^(1, *N* = 122) = 0.57, *p* = .450, Φ = .07.

**Table 4.** Firesetting behaviour variables.

Variable	Single fire(*n* = 60)				Multiple fires (*n* = 68)				Test statistic	95% CI	*p*	Effect size
	*M*	*SD*	% yes	*n*	*M*	*SD*	% yes	*n*				
Number of fires set in childhood	5.20	13.65		56	30.32	66.72		63	1168.00^a^	[−8.00, −0.00]	<.001***	−.31^d^
Age at first childhood firesetting	8.46	3.13		24	9.59	2.54		39	−1.57^b^	[−2.57, 0.31]	.122	−0.41^e^
Age at last firesetting	29.10	10.57		49	27.65	8.37		62	1538.50^a^	[−2.00, 4.00]	.910	−.01^d^
Denies any firesetting incident			18.87	10			25.00	16	0.33^c^		.568	0.05^f^
Any cell fires			20.00	12			57.35	39	17.03^c^		<.001***	0.36^f^
Any self-directed firesetting			16.95	10			27.27	18	1.36^c^		.243	0.10^f^
Any face-to-face violence via firesetting			16.07	9			13.43	9	0.02^c^		.876	0.01^f^
Any indirect violence via firesetting			14.29	8			21.21	14	0.57^c^		.450	0.07^f^

Note: CI = confidence interval.

^a^Assumption of normality violated: Mann–Whitney *U* test. ^b^*t* test. ^c^Chi-square test used.

^d^Effect size measure: *r*. ^e^Effect size measure: Cohen’s *d*. ^f^Effect size measure: phi.

***p < .001.

### Psychological vulnerabilities

Table 5 shows the correlations between total scores and subscale scores on each psychometric measure and the number of self-reported fires set in adulthood. The majority of these correlations were small and did not reach statistical significance. However, there were significant correlations between the number of fires set and the Total Score of the Four Factor Fire Scales (*r* = .21, *p* = .015), the Identification with Fire subscale score (*r* = .33, *p* < .001) and the MCAA–Part B Entitlement subscale score (*r* = .18, *p* = .048).

**Table 5.** Correlations between psychological vulnerability assessment scores and number of self-reported fires set in adulthood.

Treatment need assessment	*n*	*M*	*SD*	*r*	95% CI	*p*
Fire related measures						
FFFS						
FFFS Total Score	128	43.13	9.40	.21	[0.04, 0.37]	.015*
FFFS Identification with Fire Score	128	32.39	14.62	.33	[0.17, 0.48]	.001**
FFFS Serious Fire Interest	128	24.37	11.58	.14	[−0.04, 0.30]	.123
FFFS Poor Fire Safety Score	128	34.42	8.90	.03	[−0.14, 0.20]	.719
FFFS Firesetting as Normal Score	128	61.2	15.6	−.08	[−0.25, 0.09]	.354
Offence supportive attitude measures						
MCAA–Part B						
MCAA–Part B Total Score	128	25.92	10.11	.17	[−0.00, 0.34]	.051
MCAA–Part B Violence Score	128	5.54	3.37	.13	[−0.05, 0.29]	.156
MCAA–Part B Entitlement Score	128	6.54	2.72	.18	[0.00, 0.34]	.048*
MCAA–Part B Antisocial Score	128	6.54	3.73	.14	[−0.04, 0.30]	.125
MCAA–Part B Associates Score	128	7.30	2.47	.12	[−0.06, 0.28]	.193
Self and emotional regulation measures						
NAS–PI						
NAS Total Score	128	59.29	11.62	.05	[−0.13, 0.22]	.611
NAS Cognitive Score	128	60.84	12.23	.02	[−0.16, 0.19]	.847
NAS Arousal Score	128	57.80	14.28	.05	[−0.12, 0.22]	.568
NAS Behavioural Score	128	59.23	11.38	.06	[−0.12, 0.23]	.525
NAS Anger Regulation Score	128	46.83	12.54	.01	[−0.16, 0.18]	.913
PI Score	128	53.11	11.77	−.01	[−0.18, 0.17]	.959
Locus of Control	73	25.32	5.86	.03	[−0.20, 0.26]	.772
BIS						
BIS Total Score	55	76.29	11.82	−.10	[−0.35, 0.17]	.472
BIS Attentional Impulsiveness Score	55	19.45	4.28	−.12	[−0.38, 0.15]	.370
BIS Motor Impulsiveness Score	55	27.15	5.09	−.05	[−0.31, 0.22]	.726
BIS Non-planning Impulsiveness Score	55	29.69	4.90	−.08	[−0.34, 0.19]	.558
CSI						
CSI Total Score	55	51.45	6.37	.03	[−0.24, 0.29]	.843
CSI Problem-Focused Engagement Score	55	13.45	2.69	−.08	[−0.34, 0.19]	.573
CSI Problem-Focused Disengagement Score	55	12.53	3.60	.15	[−0.12, 0.40]	.269
CSI Emotion-Focused Engagement Score	55	11.15	3.14	−.17	[−0.42, 0.10]	.202
CSI Emotion-Focused Disengagement Score	55	14.35	2.69	.14	[−0.13, 0.39]	.298
CSI Engagement Score	55	24.58	4.78	−.16	[−0.41, 0.11]	.248
CSI Disengagement Score	55	26.87	5.69	.16	[−0.11, 0.41]	.232
Social competence measures						
The Revised UCLA Loneliness Scale	72	42.63	11.36	.10	[−0.13, 0.33]	.389
The Simple Rathus Assertiveness Schedule	69	71.8	15.79	−.06	[−0.30, 0.18]	.603
CFSEI						
CFSEI General Scale Score	73	9.72	4.02	.10	[−0.13, 0.33]	.380
CFSEI Social Scale Score	73	5.32	2.07	−.01	[−0.24, 0.22]	.952
CFSEI Personal Scale Score	73	4.05	2.50	.12	[−0.11, 0.34]	.300
ASQ						
ASQ Total Score	55	144.24	15.77	.26	[−0.01, 0.49]	.060
ASQ Confidence Score	55	29.29	5.69	.06	[−0.21, 0.32]	.671
ASQ Discomfort Score	55	44.13	7.31	.19	[−0.08, 0.43]	.169
ASQ Relationships Score	55	20.96	5.85	−.07	[−0.33, 0.20]	.617
ASQ Approval Score	55	22.05	6.20	.13	[−0.13, 0.38]	.333
ASQ Preoccupation Score	55	27.80	7.21	.26	[−0.00, 0.49]	.053

Note: CI = confidence interval; FFFS = Four Factor Fire Scales; MCAA–Part B = Measure of Criminal Attitudes and Associates Part B; NAS = Novaco Anger Scale; PI = Provocation Inventory; BIS = Barratt Impulsiveness Scale; CSI = Coping Strategies Inventory; CFSEI = The Culture-Free Self Esteem Inventory; ASQ = Attachment Style Questionnaire; UCLA = University of California, Los Angeles.

*p < .05. **p < .01.

As can be seen in Table 6, multiple-fire individuals scored significantly higher on the Four Factor Fire Scales total score (*U* = 1391.00, *p* = .002, *r* = −.27) and the Identification with Fire subscale (*U* = 1345.00, *p* = .001, *r* = −.30). The difference between the groups’ Serious Fire Interest scores was not significant (*U* = 1691.00, *p* = .096, *r* = −.15). In a departure from our pre-registered analyses, we also examined Serious Fire Interest as a dichotomous variable (problematic; non-problematic) as this is how the construct has been considered in previous studies. This dichotomised variable was calculated using the problematic cut-off score for imprisoned males, as determined by Ó Ciardha, Tyler, et al. (2015). Scores of 19 or greater were categorised as problematic. However, there was still no significant association between multiple firesetting and Serious Fire Interest, χ^2^(1, *N* = 128) = 0.40, *p* = .526, Φ = 0.06.

**Table 6.** Differences in psychological vulnerability assessment scores between single-fire and multiple-fire individuals.

Treatment need assessment	Cohort sample size	Single fire*n* = 60			Multiple fires *n* = 68						
		*M*	*SD*	*n*	*M*	*SD*	*n*	Test statistic	95% CI	*p*	Effect size
Fire related measures											
FFFS											
FFFS Total Score	128	40.29	7.55	60	45.63	10.19	68	1391.00^a^	[−8.07, −1.87]	.002**	−.27^c^
FFFS Identification with Fire Score	128	27.36	10.66	60	36.82	16.21	68	1345.00^a^	[−12.76, −1.82]	.001**	−.30^c^
FFFS Serious Fire Interest Score	128	22.02	9.37	60	26.45	12.94	68	1691.00^a^	[−7.29, 0.01]	.096	−.15^c^
FFFS Poor Fire Safety Score	128	32.88	8.51	60	35.78	9.08	68	−1.86^b^	[−6.00, 0.19]	.065	−0.33^d^
FFFS Firesetting as Normal Score	128	58.37	15.36	60	63.70	15.50	68	−1.95^b^	[−10.70, 0.08]	.053	−0.35^d^
Offence supportive attitude measures											
MCAA–Part B											
MCAA–Part B Total Score	128	24.30	10.11	60	27.35	9.97	68	−1.72^b^	[−6.57, 0.47]	.088	−0.30^d^
MCAA–Part B Violence Score	128	4.99	3.83	60	6.01	3.68	68	1700.50^a^	[−2.00, 0.00]	.104	−.14^c^
MCAA–Part B Entitlement Score	128	6.21	2.62	60	6.84	2.77	68	−1.31^b^	[−1.58, 0.32]	.191	−0.23^d^
MCAA–Part B Antisocial Score	128	6.15	3.63	60	6.88	3.80	68	1794.50^a^	[−2.00, 0.67]	.240	−.10^c^
MCAA–Part B Associates Score	128	6.95	2.45	60	7.62	2.45	68	1626.00^a^	[−1.00, −0.00]	.045*	−.18^c^
Self and emotional regulation measures											
NAS–PI											
NAS Total Score	128	57.08	11.30	60	61.24	11.62	68	1593.50^a^	[−8.67, −0.33]	.033*	−.19^c^
NAS Cognitive Score	128	55.58	11.94	60	62.82	12.22	68	1563.00^a^	[−9.00, −0.00]	.023*	−.20^c^
NAS Arousal Score	128	54.88	13.81	60	60.38	14.28	68	1578.00^a^	[−11.00, −0.00]	.023*	−.19^c^
NAS Behavioural Score	128	57.77	11.37	60	60.51	11.32	68	1766.00^a^	[−7.00, 2.00]	.191	−.12^c^
NAS Anger Regulation Score	128	48.75	13.29	60	45.13	11.67	68	2405.00^a^	[−0.00, 9.00]	.081	−.15^c^
PI Score	128	52.48	11.75	60	53.66	11.85	68	1833.50^a^	[−6.00, 2.00]	.325	−.15^c^
Locus of Control	73	25.45	5.42	46	25.11	6.64	27	0.24^b^	[−2.51, 3.19]	.815	0.06^d^
BIS											
BIS Total Score	55	69.57	10.84	14	78.59	11.37	41	−2.59^b^	[−16.00, −2.04]	.012*	−0.80^d^
BIS Attentional Impulsiveness Score	55	17.57	4.55	14	20.10	4.04	41	−1.96^b^	[−5.12, 0.06]	.056	−0.61^d^
BIS Motor Impulsiveness Score	55	24.46	3.65	14	28.00	5.27	41	−2.20^b^	[−6.41, −0.30]	.032*	−0.68^d^
BIS Non-planning Impulsiveness Score	55	27.36	4.07	14	30.49	4.95	41	−2.13^b^	[−6.08, −0.18]	.038*	−0.66^d^
CSI											
CSI Total Score	55	51.43	6.00	14	51.46	6.57	41	337.50^a^	[−2.00, 4.00]	.333	−.13^c^
CSI Problem-Focused Engagement Score	55	13.93	2.09	14	13.27	2.87	41	0.79^b^	[−1.02, 2.34]	.433	0.24^d^
CSI Problem-Focused Disengagement Score	55	11.86	3.90	14	12.76	3.52	41	−0.80b	[−3.14, 1.35]	.426	−0.25^d^
CSI Emotion-Focused Engagement Score	55	11.93	3.20	14	10.88	3.11	41	348.00^a^	[−1.00, 3.00]	.239	−.16^c^
CSI Emotion-Focused Disengagement Score	55	13.71	2.81	14	14.56	2.65	41	−1.02^b^	[−2.52, 0.82]	.314	−0.32^d^
CSI Engagement Score	55	25.86	4.70	14	24.15	4.78	41	1.16^b^	[−1.25, 4.67]	.251	0.36^d^
CSI Disengagement Score	55	25.57	6.37	14	27.32	5.45	41	−0.99^b^	[−5.28, 1.79]	.326	−0.31^d^
Social competence measures											
The Revised UCLA Loneliness Scale	73	43.01	10.58	45	42.00	12.74	27	619.00^a^	[−5.84, 7.00]	.898	−.02^c^
The Simple Rathus Assertiveness Schedule	73	71.85	14.92	43	71.54	17.42	26	0.11^b^	[−7.47, 8.30]	.917	0.03^d^
CFSEI											
CFSEI General Scale Score	73	9.82	3.94	46	9.56	4.21	27	645.00^a^	[−2.00, 2.00]	.787	−.03^c^
CFSEI Social Scale Score	73	5.24	2.17	46	5.44	1.93	27	604.00^a^	[−1.00, 1.00]	.847	−.02^c^
CFSEI Personal Scale Score	73	4.15	2.47	46	3.89	2.59	27	655.00^a^	[−1.00, 2.00]	.699	−.05^c^
ASQ											
ASQ Total Score	55	142.14	19.55	14	144.95	14.48	41	−0.57^b^	[−12.70, 7.05]	.570	−0.18^d^
ASQ Confidence Score	55	29.21	5.04	14	29.32	5.95	41	−0.06^b^	[−3.67, 3.46]	.954	−0.02^d^
ASQ Discomfort Score	55	42.43	7.84	14	44.71	7.12	41	−1.01^b^	[−6.81, 2.26]	.318	−0.31^d^
ASQ Relationships Score	55	19.36	5.93	14	21.51	5.79	41	225.00^a^	[−6.00, 1.00]	.233	−.16^c^
ASQ Approval Score	55	22.79	4.87	14	21.80	6.63	41	0.51^b^	[−2.90, 4.86]	.614	0.16^d^
ASQ Preoccupation Score	55	28.36	6.61	14	27.61	7.48	41	0.33^b^	[−3.77, 5.26]	.741	0.10^d^

Note: CI = confidence interval; FFFS = Four Factor Fire Scales; MCAA–Part B = Measure of Criminal Attitudes and Associates Part B; NAS = Novaco Anger Scale; PI = Provocation Inventory; BIS = Barratt Impulsiveness Scale; CSI = Coping Strategies Inventory; CFSEI = The Culture-Free Self Esteem Inventory; ASQ = Attachment Style Questionnaire; UCLA = University of California, Los Angeles.

^a^Assumption of normality violated: Mann–Whitney *U* test used. ^b^*t* test used. ^c^Effect size measure: *r*. ^d^Effect size measure: Cohen’s *d.*

*p < .05. **p < .01.

In terms of offence supportive measures, the only significant difference was demonstrated on the MCAA–Part B Associates subscale (*U* = 1626.00, *p =* .045, *r* = −.18), with multiple-fire individuals scoring higher.

On self and emotional regulation measures, the groups demonstrated significant differences on two subscales of the NAS. Multiple-fire individuals scored significantly higher on the Cognitive subscale (*U* = 1563.00, *p* = .023, *r* = −.20) and the Arousal subscale (*U* = 1578.00, *p* = .023, *r* = −.19). Furthermore, on the NAS Total Score, multiple-fire individuals scored significantly higher than single-fire individuals (*U* = 1593.50, *p* = .033, *r* = −.19). In addition, multiple-fire individuals scored significantly higher on the BIS total score, *t*(53) = −2.59, *p* = .012, Cohen’s *d =* −0.81, the BIS Motor Impulsiveness subscale, *t*(53) = −2.20, *p* = .032, Cohen’s *d =* −0.68, and the BIS Non-planning Impulsiveness subscale, *t*(53) = −2.13, *p* = .038, Cohen’s *d* = −0.66. Although the difference on the BIS Attentional Impulsiveness subscale did not reach statistical significance, *t*(53) = −1.96, *p* = .056, there was still a medium effect size (Cohen’s *d =* −0.61).

The groups did not display any significant differences in their scores on any of the social competence measures (see Table 6).

Those variables that were related to measures completed by both cohorts of participants and reached statistical significance or demonstrated a medium effect size in prior analyses were initially selected for entry into a logistic regression. Psychological vulnerability variables that fulfilled these criteria were as follows: FFFS Total Score; FFFS Identification with Fire Score; MCAA Entitlement Score; MCAA Associates Score; NAS Total Score; NAS Cognitive Score; NAS Arousal Score. Potential covariates that fulfilled the inclusion criteria were as follows: Number of fires set in childhood; Any cell fires; Number of convictions; Number of convicted offences; Number of theft and kindred offences; Number of offences relating to Police, Courts and Prison; Number of firearms offences; Number of miscellaneous offences; Number of cautions; Number of cautionable offences; Number of violent non-sexual offences.

However, due to the fact that this high number of variables would violate Vittinghoff and McCulloch (2007) guidance on minimum EPV, and the high multi-collinearity between the variables (variance inflation factor, VIF scores ranging from 1.15 to 52.82), the variables that were entered into the model were narrowed. Specifically, rather than including individual offence types, only the higher level variables of the number of convicted offences and number of cautionable offences were entered. In addition, the NAS Total Score was also excluded due to high multi-collinearity with the selected NAS subscales. The FFFS Total Score was retained as VIF scores all remained below 5 with this in the model. See Table 7 for included variables.

**Table 7.** Logistic regression model.

Variable	*B*	*SE*	*p*	*OR*	95% CI
FFFS Total Score	−0.10	0.06	.161	0.91	[0.80, 1.02]
FFFS Identification with Fire Score	0.08	0.04	.022*	1.09	[1.01, 1.17]
MCAA–Part B Entitlement Score	−0.12	0.11	.295	0.89	[0.71, 1.10]
MCAA–Part B Associates Score	0.11	0.13	.415	1.11	[0.86, 1.44]
NAS Cognitive Score	0.02	0.04	.684	1.02	[0.94, 1.10]
NAS Arousal Score	0.05	0.03	.140	1.05	[0.99, 1.11]
Covariates					
Number of childhood fires	0.02	0.01	.112	1.02	[1.00, 1.05]
Cell fires	1.92	0.55	<.001***	6.83	[2.42, 21.45]
Number of convicted offences	0.00	0.01	.580	1.00	[0.99, 1.02]
Number of cautionable offences	0.34	0.17	.046*	1.40	[1.01, 1.98]

Note: CI = confidence interval; *OR* = odds ratio; FFFS = Four Factor Fire Scales; MCAA–Part B = Measure of Criminal Attitudes and Associates Part B; NAS = Novaco Anger Scale. *R*^2^ = .327 (Hosmer–Lemeshow), .364 (Cox–Snell), .486 (Nagelkerke). Model χ^2^(10) = 50.30, *p <* .001.

*p < .05. ***p < .001.

The full model was statistically significant, χ^2^(10) = 50.30, *p* < .001. The model explained between 32.7% (Hosmer–Lemeshow *R*^2^) and 48.6% (Nagelkerke *R*^2^) of the variance in frequency of firesetting behaviour. The model correctly classified 81.25% of cases: 87.5% of multiple-fire individuals and 75.0% of single-fire individuals. ROC analyses demonstrated that the model effectively discriminated between single-fire and multiple-fire individuals (AUC = .86, 95% confidence interval, CI [.72, .99]). As reported in Table 7, the only psychological vulnerability variable that made a unique statistically significant contribution to the model was the FFFS Identification with Fire subscale. In terms of background factors that were entered as covariates, a history of setting cell fires (*p* < .001; odds ratio, *OR* = 6.83) and number of cautionable offences (*p* = .046, *OR* = 1.40) made unique statistically significant contributions to the model. No observations had a Cook’s distance greater than 1, so no outliers were removed from the analyses.

## Discussion

This is the first study to take a theoretically informed approach to the examination of dynamic risk factors hypothesised to be associated with multiple firesetting, using validated psychometric measures. We investigated factors falling within each of the four domains of psychological vulnerability hypothesised by the M-TTAF, and found evidence supporting fire-related factors, general offence supportive attitudes and self-regulation issues as potential dynamic risk factors for multiple firesetting. We also found a number of offence history and firesetting behaviour variables were associated with setting multiple fires.

### Background factors

To capture factors that may co-vary with the psychological vulnerabilities, we first examined several background variables, relating to demographics and offence histories. In contrast to Ó Ciardha, Barnoux, et al. (2015) and Sapsford et al. (1978), we did not find a significant difference between single-fire and multiple-fire individuals in terms of their sentence length. It should be noted, however, that this variable was not available for all participants, as some were on remand and had not yet been convicted or sentenced for their index offence. We also did not find a significant difference in terms of whether participants had ever received a psychiatric diagnosis. This is inconsistent with previous research that has found an association between repeat firesetting and psychiatric diagnoses (e.g. Dickens et al., 2009; Ducat et al., 2015; Rice & Harris, 1991). While the current study used a sample recruited from prisons, psychiatric diagnoses were still prevalent, although in line with rates in prison populations more widely (see Tyler, Miles, et al., 2019).

Consistent with prior research (e.g. Ducat et al., 2015), we found a number of differences between the single-fire and multiple-fire individuals in terms of their offence histories. Multiple-fire individuals had more prolific criminal records, with significantly greater numbers of convictions in several offence categories, and the number of cautionable offences made a significant contribution to the logistic regression model. These findings provide further evidence that individuals with a history of firesetting engage in a variety of criminal activities, and suggest that wider antisocial behaviour may co-occur alongside persistent firesetting. Consequently, firesetting risk assessments should incorporate information about an individual’s broader offending. However, the groups did not significantly differ in terms of the number of convictions for firesetting offences. This emphasises the importance of not solely relying on official sources of firesetting in risk assessments as this can result in an underestimation of reoffending (see Sambrooks, 2021; Sambrooks et al., 2021).

### Firesetting behaviour

Consistent with previous research, we found individuals who had set multiple fires in adulthood had also set significantly more fires during childhood (Edwards & Grace, 2014; Rice & Harris, 1991, 1996; Tyler et al., 2015). Therefore, early prevention strategies for youth at risk of engaging in firesetting behaviour are of vital importance to reduce the risk of persistent firesetting into adulthood. Previous research has suggested that multiple-fire individuals are likely to have begun firesetting at an earlier age (Ducat et al., 2015; Edwards & Grace, 2014; Rice & Harris, 1991, 1996). In the current research, however, the difference between single-fire individuals and multiple-fire individuals in terms of their age at their first childhood firesetting incident did not reach statistical significance.

We also investigated variables relating to the context of participants’ firesetting. A history of setting fires within prison was the only variable that was associated with multiple firesetting. It was also the variable that made the largest contribution to our regression model; participants who had set a cell fire had almost seven times greater odds of having set multiple fires. There is a dearth of literature regarding firesetting within institutional settings, despite it being a prevalent problem across prisons and secure psychiatric hospitals (Willmot & Mason, 2023). For example, in the year to April 2021, 91% of the 1,003 fires reported within prison establishments in England and Wales were determined to have been deliberately set (Home Office, 2022a). While this cross-sectional research is unable to determine whether cell fires are predictive of multiple firesetting, it is clear that clinicians need to be cognisant of institutional firesetting in their risk assessments and treatment planning. In addition to its association with multiple firesetting, it is important to note that 20% of single-fire individuals reported having set a cell fire, indicating that their only firesetting experience has been within prison. This suggests that for some individuals being imprisoned may represent a proximal trigger that exacerbates their psychological vulnerabilities to a threshold that results in them engaging in deliberate firesetting (see Gannon et al., 2012). This aligns with recent research examining institutional firesetting that found that only 16% of individuals who had set fires within prisons or psychiatric settings had convictions for firesetting in the community (Willmot & Mason, 2023). Further, the likelihood of being prosecuted for institutional firesetting is very low, with only around 10.5% of institutional firesetting incidents resulting in a criminal conviction (Willmot & Mason, 2023). The Crown Prosecution Service explicitly states that in cases where the cell fire may be an attempt to self-harm, prosecutions should not be sought (Crown Prosecution Service, 2023). Therefore, it is crucial that both academics and clinicians within the prison estate and other institutional settings consider wider reports of firesetting, not just convictions, when considering the risk of repeat firesetting.

### Fire-related factors

Psychometric measures tapping into the four domains of psychological vulnerability hypothesised by the M-TTAF were examined. In terms of fire-related risk factors, the M-TTAF suggests that holding an inappropriate interest in fire is associated with firesetting (Gannon et al., 2012). In support of this, one of the most consistent findings in the prior literature examining firesetting risk factors is an association between increased fire interest and repeat firesetting (e.g. MacKay et al., 2006; Tyler et al., 2015). Therefore, we hypothesised that individuals who had set multiple fires would score significantly higher on the FFFS Serious Fire Interest subscale than individuals who had only set one fire. However, the difference in scores failed to reach statistical significance, and only a small effect size was reported. There was also no significant correlation between scores on this subscale and the number of self-reported fires set in adulthood.

This was surprising given the results of the previous studies, particularly Tyler et al.’s (2015) finding that fire interest was the largest unique predictor of repeat firesetting among psychiatric patients, with an odds ratio exceeding 15. However, it is important to note that there are a number of methodological differences between Tyler et al.’s study and the current research. While the current study used the presently recommended psychometric measure for assessing inappropriate fire interest (the FFFS; see Gannon et al., 2022) and therefore measured the construct in a standardised way, Tyler and colleagues coded the presence of fire interest from proxy indicators that were detailed in patients’ clinical notes, with little information available regarding how this interest was initially judged. The FFFS typically measures fire interest on a continuum, determined from several questions assessing the construct, whereas Tyler et al. considered fire interest as a dichotomised variable – inappropriate fire interest was either present in patients’ clinical notes or not. It is possible that where fire interest was coded as absent, the individual may have held an interest in fire but it had not been explored or assessed, and was therefore absent from their clinical notes. Alternatively, Tyler et al.’s operationalisation may represent a higher threshold of fire interest, since for fire interest indicators to be recorded in a patient’s notes it is likely to have translated to their behaviour or speech. Therefore, this dichotomisation may be making a more meaningful distinction between a level of fire interest that is associated with multiple firesetting and a level that is inconsequential for firesetting behaviour. However, when we dichotomised FFFS Serious Fire Interest scores into problematic and non-problematic scores in order to generate a meaningful distinction on levels of fire interest (according to Ó Ciardha, Tyler, et al.’s, 2015, cut-off scores), there was still no significant difference between single-fire and multiple-fire individuals in terms of the proportion of participants whose scores were problematic.

Another potentially important difference to note is that Tyler et al.’s (2015) sample were patients recruited from psychiatric facilities, whereas the current study used an imprisoned sample. This may be an important distinction because previous research has suggested that individuals with a history of firesetting should not be considered a homogeneous group in terms of their treatment needs (Ó Ciardha, Tyler, et al., 2015). In particular, there are significant differences between the scores of imprisoned samples and psychiatric samples on the FFFS (Ó Ciardha, Tyler, et al., 2015). Thus, the lack of consistency between the current study and Tyler et al.’s findings may be due to differences in the importance of fire interest in terms of its influence on risk for repeat firesetting across the two sample types. Indeed, the findings of the current study are consistent with previous research using the FFFS with a prison-based sample; Ó Ciardha, Barnoux, et al. (2015) established that the FFFS Serious Fire Interest subscale did not accurately discriminate between imprisoned males with single and multiple firesetting incidents. Consequently, future research should endeavour to examine the association between inappropriate fire interest and repeat firesetting across a range of populations.

In terms of the other subscales of the FFFS, our findings again align with those of Ó Ciardha, Barnoux, and colleagues (2015), in that the Identification with Fire subscale was the only subscale to demonstrate a significant difference between single-fire individuals and multiple-fire individuals. Those who had set multiple fires reported more agreement with statements suggesting fire is an essential part of their functioning. This was also the only psychological vulnerability measure that made a significant unique contribution to the logistic regression model. Even when controlling for childhood firesetting, setting of cell fires and the number of offences recorded on the PNC, identification with fire scores significantly predicted the categorisation of participants as multiple-fire individuals. Thus, addressing an individual’s fire-specific treatment needs, and particularly their affinity with fire, through specialist interventions is likely to be an important avenue for attempting to reduce the likelihood of persistent firesetting. In addition, using the FFFS to screen for identification with fire may be a useful strategy for prioritising individuals for treatment or for identifying those with an increased probability of future firesetting in risk assessments.

### Offence supportive attitudes

There was little evidence that fire-specific offence supportive attitudes were more prevalent among individuals who had set multiple fires than among those who had set only one fire. However, it should be noted that there are other aspects of firesetting-related cognition that are not explicitly assessed by the FFFS: for example, implicit theories (see Ó Ciardha & Gannon, 2012) or inappropriate fire scripts (see Butler & Gannon, 2015). Consequently, it is possible that there may be other fire-specific cognitive elements that are more prevalent among individuals who set multiple fires that have not yet been investigated. Recently, a new measure that incorporates assessment of inappropriate fire scripts has been developed (Gannon et al., 2023), which presents an opportunity to conduct further research examining the association between these previously overlooked aspects of firesetting cognition and multiple firesetting.

There was some support for general offence supportive attitudes playing a role in multiple firesetting. Multiple-fire individuals scored significantly higher on the MCAA–Part B Associates subscale, indicating that they hold more attitudes that are favourable towards having antisocial friends (Mills et al., 2004). Meanwhile, scores on the MCAA–Part B Entitlement subscale were significantly positively correlated with the number of fires set in adulthood. Neither of these subscales were uniquely significant in the logistic regression model. However, both the number of convicted offences and the number of cautionable offences recorded on the PNC were entered into the model as covariates, with the latter reaching statistical significance (*OR* = 1.40). These covariates are likely also tapping into the individual’s inclination to wider antisociality and thus may explain why the MCAA scores failed to make a unique significant contribution to the model.

### Self and emotional regulation issues

Individuals who had set multiple fires differed from single-fire individuals on several of the measures of self and emotional regulation issues. In particular, multiple-fire individuals showed greater anger justification and rumination and held more hostile attitudes (NAS–PI Cognitive subscale). They also exhibited greater anger intensity and higher levels of irritability (NAS–PI Arousal subscale) than single-fire individuals. These findings are perhaps unsurprising given the well-established prevalence of aggressive motives for firesetting (see Doley et al., 2011). However, they do somewhat contrast with Rice and Harris’s (1991, 1996) research, which showed that patients who engaged in repeat firesetting were less likely to have a history of interpersonal aggression than patients who had set only one fire. While Rice and Harris (1991) provide little information on how they assessed this variable, given that they explicitly referred to a history of aggression, it is likely they utilised behavioural reports. In contrast, the current study focused primarily on psychometric measures of the cognition and affect underlying aggression. However, this methodological difference alone is unlikely to fully account for the disparity in findings, since this study found that PNC records of violent offences indicated that multiple-fire individuals engaged in significantly more aggressive acts. Further research utilising both psychometric and behavioural measures of aggression is needed to better determine its influence on repeat firesetting.

All subscales of the BIS reached either statistical significance or a medium effect size, indicating greater levels of impulsivity among individuals who have set multiple fires. This is consistent with Wyatt’s (2018) finding that the odds of psychiatric patients setting multiple fires increased threefold if they were known to be impulsive. However, in Wyatt’s research impulsivity was coded as present or absent from patients’ hospital notes. While these clinical notes were reported to include psychological assessments, no details on the assessment tools used were provided. In the current study, BIS scores were not entered into the logistic regression model due to the BIS only being completed by the Sambrooks and Tyler (2019) cohort. As a result, whether scores on the BIS represent a dynamic risk factor for multiple firesetting is currently unknown.

### Social competence issues

There were no significant differences between individuals who had only set one fire and individuals who had set multiple fires in terms of loneliness, assertiveness, self-esteem or attachment style. However, since Gannon et al. (2013) found that the measures of loneliness and assertiveness failed to distinguish between firesetting and non-firesetting individuals, the battery of social competence measures was updated for the Sambrooks and Tyler (2019) cohort. This resulted in smaller sample sizes across these measures, and consequently there was only sufficient power to detect much larger differences between the groups. Future research should endeavour to investigate differences in social competence using larger samples informed by a priori power analyses.

## Limitations

As already discussed, our conclusions are constrained by the sample sizes used, particularly where variables were recorded for only one cohort of participants. In addition, since our samples were recruited from prison establishments, the findings may not be reflective of firesetting adults in other settings. It is well established that deliberate firesetting is also a prevalent issue in both hospital settings and the community (see Gannon et al., 2022), and therefore dynamic risk factors for repeat firesetting in individuals residing in these settings still need to explored. Our sample was also exclusively male. While deliberate firesetting does appear to be more prevalent among males, it is still a significant issue among females (Nanayakkara et al., 2020), with women estimated to be responsible for 15–20% of deliberate fires (Ducat et al., 2013; Gannon, 2010). Therefore, further research is vital to investigate whether the findings of this study extend to other populations with a history of firesetting.

Another potential limitation stems from the variable used to categorise individuals on the basis of their firesetting behaviour. We only had information available on the number of fires set in adulthood, rather than the number of firesetting incidents, meaning it was not possible to determine whether those individuals who had set multiple fires engaged in repeat firesetting, or if all their fires had been set in one incident. Therefore, the findings have limited utility for directly informing risk assessments that are primarily concerned with whether individuals will engage in further incidents of firesetting. However, self-report data on the number of fires set were deemed to be more appropriate than utilising the number of convictions for firesetting offences because, as already mentioned, there is often a significant disparity between official records of the legal offence of arson and other indicators of deliberate firesetting. For example, there were 898 individuals sentenced for arson during 2015 across both England and Wales (Sentencing Council, 2022), despite English Fire and Rescue Services attending 73,674 deliberate fires in the same year (Home Office, 2022b). Indeed, 31.3% of the current sample (*n =* 40) had not received a conviction for a firesetting offence. We believe that utilising self-report data on the number of fires set therefore provides a more accurate picture of firesetting behaviour among imprisoned individuals.

Finally, we urge caution when interpreting the results of the univariate analyses independently given that no correction to significance was implemented. Furthermore, the cross-sectional nature of this study means that our results reflect differences between single-fire and multiple-fire individuals only at a single point in time. We are unable to determine whether there is a predictive relationship between the variables studied and multiple firesetting. Prospective longitudinal research is needed before these differences can be used as evidence to inform risk assessments.

## Conclusions

This study is the first to investigate whether psychometric measures of the psychological vulnerabilities outlined in the M-TTAF distinguish between individuals who have set a single deliberate fire and individuals who have set multiple fires. Our findings provide evidence that, even when controlling for previous recorded offences and firesetting behaviour variables, increased levels of fire-specific treatment needs (particularly identification with fire) play a role in persistent firesetting and therefore need to be targeted as part of assessment and interventions. In addition, cognition related to anger and general offence supportive attitudes should be targeted, alongside irritability and impulsiveness. Examination of firesetting behaviour variables emphasise the importance of early prevention strategies and close monitoring of individuals who have set cell fires. Future studies should adopt longitudinal designs to ensure that the covariation between the factors identified in this study and repeat firesetting is prospective and to provide clear evidence that they represent true dynamic risk factors for the setting of multiple fires (Bonta & Andrews, 2016).

## Ethical standards

### Declaration of conflicts of interest

Katie Sambrooks has declared no conflicts of interest.

Nichola Tyler has declared no conflicts of interest.

Theresa A. Gannon has declared no conflicts of interest.

### Ethical approval

All procedures performed in studies involving human participants were in accordance with the ethical standards of the institutional and/or national research committee (University of Kent Research Ethics Committee [Ref 20101507; Ref 201815434893195257] and the National Offender Management Service Research Committee [Ref 74-10; Ref 2018-385]) and with the 1964 Helsinki declaration and its later amendments or comparable ethical standards.

### Informed consent

Informed consent was obtained from all individual participants included in the study.

## Footnotes

1 Their index offence was not necessarily fire-related.

2 The number of fires set before 18 years old were excluded from this categorisation.

3 This included any fire deliberately set within a prison establishment.

4 This was defined as using fire to deliberately harm someone while being face to face with them.

5 This was defined as using fire to deliberately harm someone but without being face-to-face.

6 Due to only having total scores for each subscale and not responses for each individual item in measures completed by the Gannon et al. (2015) cohort, we were unable to compute reliability statistics in the current study for the majority of measures.

7 This included thefts, burglaries, robberies, and proceeds of crime offences.

## References

[CIT0001] Addison, C. C., Campbell-Jenkins, B. W., Sarpong, D. F., Kibler, J., Singh, M., Dubbert, P., Wilson, G., Payne, T., & Taylor, H. (2007). Psychometric evaluation of a Coping Strategies Inventory Short-Form (CSI-SF) in the Jackson Heart Study Cohort. *International Journal of Environmental Research and Public Health*, *4*(4), 289–295. 10.3390/ijerph200704040004 18180539 PMC3732399

[CIT0002] Andrews, D. A., & Bonta, J. (2010). *The psychology of criminal conduct*. Routledge.

[CIT0003] Battle, J. (1992). Culture-free self-esteem inventories: Examiner’s manual (2nd ed.). PRO-ED.

[CIT0004] Battle, J. (1997). Culture-free self-esteem inventories for children and adults. In C. P. Zalaquett & R. J. Wood (Eds.), *Evaluating stress: A book of resources* (pp. 67–95). The Scarecrow Press, Inc.

[CIT0005] Bell, R., Doley, R. M., & Dawson, D. (2018). Developmental characteristics of firesetters: Are recidivist offenders distinctive? *Legal and Criminological Psychology*, *23*(2), 163–175. 10.1111/lcrp.12135

[CIT0006] Bonta, J., & Andrews, D. A. (2016). *The psychology of criminal conduct* (6th ed.). Routledge. 10.4324/9781315677187

[CIT0007] Butler, H., & Gannon, T. A. (2015). The scripts and expertise of firesetters: A preliminary conceptualization. *Aggression and Violent Behavior*, *20*, 72–81. 10.1016/j.avb.2014.12.011

[CIT0008] Campbell, R. (2021). *Intentional structure fires*. National Fire Protection Association. https://www.nfpa.org//-/media/Files/News-and-Research/Fire-statistics-and-reports/US-Fire-Problem/Fire-causes/osintentionalTables.pdf

[CIT0009] Crown Prosecution Service. (2023). *Prison-related offences.* https://www.cps.gov.uk/legal-guidance/prison-related-offences-0

[CIT0010] Culhane, S. E., & Morera, O. F. (2010). Reliability and validity of the Novaco Anger Scale and Provocation Inventory (NAS-PI) and State-Trait Anger Expression Inventory-2 (STAXI-2) in Hispanic and Non-Hispanic white student samples. *Hispanic Journal of Behavioral Sciences*, *32*(4), 586–606. 10.1177/0739986310381458

[CIT0011] Dickens, G. L., Sugarman, P., Edgar, S., Hofberg, K., Tewari, S., & Ahmad, F. (2009). Recidivism and dangerousness in arsonists. *Journal of Forensic Psychiatry & Psychology*, *20*(5), 621–639. 10.1080/14789940903174006

[CIT0012] Doley, R. M., Fineman, K., Fritzon, K., Dolan, M., & McEwan, T. E. (2011). Risk factors for recidivistic arson in adult offenders. *Psychiatry, Psychology and Law*, *18*(3), 409–423. 10.1080/13218719.2011.559155

[CIT0013] Ducat, L., McEwan, T. E., & Ogloff, J. R. (2015). An investigation of firesetting recidivism: Factors related to repeat offending. *Legal and Criminological Psychology*, *20*(1), 1–18. 10.1111/lcrp.12052

[CIT0014] Ducat, L., Ogloff, J. R., & McEwan, T. (2013). Mental illness and psychiatric treatment amongst firesetters, other offenders and the general community. *The Australian and New Zealand Journal of Psychiatry*, *47*(10), 945–953. 10.1177/0004867413492223 23739314

[CIT0015] Edwards, M. J., & Grace, R. C. (2014). The development of an actuarial model for arson recidivism. *Psychiatry, Psychology and Law*, *21*(2), 218–230. 10.1080/13218719.2013.803277

[CIT0016] Federal Bureau of Investigation. (2022). *Crime data explorer.* https://cde.ucr.cjis.gov/LATEST/webapp/#/pages/explorer/crime/arrest

[CIT0017] Feeney, J. A., Noller, P., & Hanrahan, M. (1994). Assessing adult attachment. In M. B. Sperling & W. H. Berman (Eds.), *Attachment in adults: Clinical and developmental perspectives* (pp. 128–152). Guildford Press.

[CIT0018] Field, O. H. (2016). Risk factors for arson recidivism in adult offenders [PhD thesis]. University of Birmingham]. UBIRA e theses. https://etheses.bham.ac.uk/id/eprint/6639/

[CIT0019] Gannon, T. A. (2010). Female arsonists: Key features, psychopathologies, and treatment needs. *Psychiatry: Interpersonal and Biological Processes*, *73*(2), 173–189. 10.1521/psyc.2010.73.2.173 20557228

[CIT0020] Gannon, T. A. (2017). *Firesetting Intervention Programme for Prisoners (FIPP) version 2*. CORE-FP, University of Kent.

[CIT0021] Gannon, T. A., Alleyne, E., Butler, H., Danby, H., Kapoor, A., Lovell, T., Mozova, K., Spruin, E., Tostevin, T., Tyler, N., & Ó Ciardha, C. (2015). Specialist group therapy for psychological factors associated with firesetting: Evidence of a treatment effect from a non-randomized trial with male prisoners. *Behaviour Research and Therapy*, *73*, 42–51. 10.1016/j.brat.2015.07.007 26248329

[CIT0022] Gannon, T. A., Ó., Ciardha, C., Barnoux, M., Tyler, N., Mozova, & Alleyne, E. K. (2013). Male imprisoned firesetters have different characteristics than other imprisoned offenders and require specialist treatment. *Psychiatry: Interpersonal and Biological Processes*, *76*(4), 349–364. 10.1521/psyc.2013.76.4.349 24299093

[CIT0023] Gannon, T. A., Ó Ciardha, C., & Barnoux, M.-F. (2011). *The identification with fire questionnaire.* Unpublished manuscript.

[CIT0024] Gannon, T. A., Ó Ciardha, C., Doley, R. M., & Alleyne, E. (2012). The Multi-Trajectory Theory of Adult Firesetting (M-TTAF). *Aggression and Violent Behavior*, *17*(2), 107–121. 10.1016/j.avb.2011.08.001

[CIT0025] Gannon, T. A., Olver, M. E., Alleyne, E., Butler, H., Lister, V., Ó Ciardha, C., Sambrooks, K., & Tyler, N. (2023). The development and validation of the firesetting questionnaire. *Journal of Psychopathology and Behavioral Assessment*, *45*(1), 27–47. 10.1007/s10862-022-10011-x

[CIT0026] Gannon, T. A., & Pina, A. (2010). Firesetting: Psychopathology, theory and treatment. *Aggression and Violent Behavior*, *15*(3), 224–238. 10.1016/j.avb.2010.01.001

[CIT0027] Gannon, T. A., Tyler, N., Ó Ciardha, C., & Alleyne, E. (2022). *Adult deliberate firesetting: Theory, assessment and treatment*. Wiley-Blackwell.

[CIT0028] Hagenauw, L. A., Karsten, J., Akkerman-Bouwsema, G. J., de Jager, B. E., & Lancel, M. (2015). Specific risk factors of arsonists in a forensic psychiatric hospital. *International Journal of Offender Therapy and Comparative Criminology*, *59*(7), 685–700. 10.1177/0306624X13519744 24459208

[CIT0029] Home Office. (2022a). *Crown premises fire safety inspectorate annual report 2020/21.* https://assets.publishing.service.gov.uk/government/uploads/system/uploads/attachment_data/file/1105456/Final_2020-21_CPFSI_Annual_Report_.pdf

[CIT0030] Home Office. (2022b). *FIRE0401: Deliberate fires attended by the Fire and Rescue Services.* https://assets.publishing.service.gov.uk/government/uploads/system/uploads/attachment_data/file/1096220/fire-statistics-data-tables-fire0401-110822.xlsx

[CIT0031] Home Office. (2022c). *FIRE0402: Fatalities and non-fatal casualties in deliberate fires attended.* https://assets.publishing.service.gov.uk/government/uploads/system/uploads/attachment_data/file/1096222/fire-statistics-data-tables-fire0402-110822.xlsx

[CIT0032] Jenerette, C., & Dixon, J. (2010). Developing a short form of the simple Rathus assertiveness schedule using a sample of adults with sickle cell disease. *Journal of Transcultural Nursing : Official Journal of the Transcultural Nursing Society*, *21*(4), 314–324. 10.1177/1043659609360712 20592057

[CIT0033] Kennedy, P. J., Vale, E. L., Khan, S. J., & McAnaney, A. (2006). Factors predicting recidivism in child and adolescent fire-setters: A systematic review of the literature. *Journal of Forensic Psychiatry & Psychology*, *17*(1), 151–164. 10.1080/14789940500441501

[CIT0034] MacKay, S., Henderson, J., Del Bove, G., Marton, P., Warling, D., & Root, C. (2006). Fire interest and antisociality as risk factors in the severity and persistence of juvenile firesetting. *Journal of the American Academy of Child and Adolescent Psychiatry*, *45*(9), 1077–1084. 10.1097/01.chi.0000227881.50404.ca 16926615

[CIT0035] Mills, J. F., & Kroner, D. G. (1999). *Measures of Criminal Attitudes and Associates: User Guide.* Unpublished instrument and user guide.

[CIT0036] Mills, J. F., Kroner, D. G., & Forth, A. E. (2002). Measures of Criminal Attitudes and Associates (MCAA): Development, factor structure, reliability, and validity. *Assessment*, *9*(3), 240–253. 10.1177/1073191102009003003 12216781

[CIT0037] Mills, J. F., Kroner, D., & Hemmati, T. (2004). The Measures of Criminal Attitudes and Associates (MCAA): The prediction of general and violent recidivism. *Criminal Justice and Behavior*, *31*(6), 717–733. 10.1177/0093854804268755

[CIT0038] Ministry of Justice. (2022). *Criminal justice system statistics publication: Outcomes by offence pivot table analytical tool for England and Wales.* https://www.gov.uk/government/statistics/criminal-justice-system-statistics-quarterly-june-2022

[CIT0039] Muckley, A. (1997). *Firesetting: Addressing offending behaviour, a resource and training manual*. Redcar and Cleveland Psychological Service.

[CIT0040] Murphy, G. H., & Clare, I. C. (1996). Analysis of motivation in people with mild learning disabilities (mental handicap) who set fires. *Psychology, Crime and Law*, *2*(3), 153–164. 10.1080/10683169608409774

[CIT0041] Nanayakkara, V., Ogloff, J. R. P., Davis, M. R., & McEwan, T. E. (2020). Gender-based types of firesetting: Clinical, behavioural and motivational differences among female and male firesetters. *The Journal of Forensic Psychiatry & Psychology*, *31*(2), 273–291. 10.1080/14789949.2020.1720266

[CIT0042] Novaco, R. W. (2003). *The Novaco anger scale and provocation inventory: NAS-PI*. Western Psychological Services.

[CIT0043] Nowicki, S. (1976). Factor structure of locus of control in children. *The Journal of Genetic Psychology*, *129*(1st Half), 13–17. 10.1080/00221325.1976.10534005 985686

[CIT0044] Nowicki, S., & Duke, M. P. (1974). A locus of control scale for noncollege as well as college adults. *Journal of Personality Assessment*, *38*(2), 136–137. 10.1080/00223891.1974.10119950

[CIT0045] Ó Ciardha, C., Barnoux, M. F. L., Alleyne, E. K. A., Tyler, N., Mozova, K., & Gannon, T. A. (2015). Multiple factors in the assessment of firesetters’ fire interest and attitudes. *Legal and Criminological Psychology*, *20*(1), 37–47. 10.1111/lcrp.12065

[CIT0046] Ó Ciardha, C., & Gannon, T. A. (2012). The implicit theories of firesetters: A preliminary conceptualization. *Aggression and Violent Behavior*, *17*(2), 122–128. 10.1016/j.avb.2011.12.001

[CIT0047] Ó Ciardha, C., Tyler, N., & Gannon, T. A. (2015). A practical guide to assessing adult firesetters’ fire-specific treatment needs using the Four Factor Fire Scales. *Psychiatry*, *78*(4), 293–304. 10.1080/00332747.2015.1061310 26745683

[CIT0048] Patton, J. H., Stanford, M. S., & Barratt, E. S. (1995). Factor structure of the barratt impulsiveness scale. *Journal of Clinical Psychology*, *51*(6), 768–774. 10.1002/1097-4679(199511)51:6<768::AID-JCLP2270510607>3.0.CO;2-1 8778124

[CIT0049] Rice, M. E., & Harris, G. T. (1991). Firesetters admitted to a maximum security psychiatric institution: Offenders and offenses. *Journal of Interpersonal Violence*, *6*(4), 461–475. 10.1177/088626091006004005

[CIT0050] Rice, M. E., & Harris, G. T. (1996). Predicting the recidivism of mentally disordered firesetters. *Journal of Interpersonal Violence*, *11*(3), 364–375. 10.1177/088626096011003004

[CIT0051] Rice, M. E., & Harris, G. T. (2005). Comparing effect sizes in follow-up studies: ROC area, Cohen’s d, and r. *Law and Human Behavior*, *29*(5), 615–620. 10.1007/s10979-005-6832-7 16254746

[CIT0052] Russell, D., Peplau, L. A., & Cutrona, C. E. (1980). The revised UCLA loneliness scale: Concurrent and discriminant validity evidence. *Journal of Personality and Social Psychology*, *39*(3), 472–480. 10.1037/0022-3514.39.3.472 7431205

[CIT0053] Sambrooks, K. (2021). Risk assessments for deliberate firesetting: Difficulties, recent advancements, and best practice. *Assessment and Development Matters*, *13*(4), 4–7. 10.53841/bpsadm.2021.13.4.4

[CIT0054] Sambrooks, K., Olver, M. E., Page, T. E., & Gannon, T. A. (2021). Firesetting reoffending: A meta-analysis. *Criminal Justice and Behavior*, *48*(11), 1634–1651. 10.1177/00938548211013577

[CIT0055] Sambrooks, K., & Tyler, N. (2019). What works with adult deliberate firesetters? Where have we come from and where do we go from here? *Forensic Update*, *1*(130), 17–21. 10.53841/bpsfu.2019.1.130.17

[CIT0056] Sapsford, R. J., Banks, C., & Smith, D. D. (1978). Arsonists in prison. *Medicine, Science, and the Law*, *18*(4), 247–254. 10.1177/002580247801800405 703564

[CIT0057] Sentencing Council. (2022). *Arson and criminal damage: Statistical bulletin data tables.* https://www.sentencingcouncil.org.uk/publications/item/arson-and-criminal-damage-statistical-bulletin/

[CIT0058] Tyler, N., Gannon, T. A., Dickens, G. L., & Lockerbie, L. (2015). Characteristics that predict firesetting in male and female mentally disordered offenders. *Psychology, Crime & Law*, *21*(8), 776–797. 10.1080/1068316X.2015.1054382

[CIT0059] Tyler, N., Gannon, T. A., Ó., Ciardha, C., Ogloff, & Stadolnik, R. (2019). Deliberate firesetting: An international public health issue. *The Lancet Public Health*, *4*(8), e371–e372. 10.1016/S2468-2667(19)30136-7 31376854

[CIT0060] Tyler, N., Miles, H. L., Karadag, B., & Rogers, G. (2019). An updated picture of the mental health needs of male and female prisoners in the UK: Prevalence, comorbidity, and gender differences. *Social Psychiatry and Psychiatric Epidemiology*, *54*(9), 1143–1152. 10.1007/s00127-019-01690-1 30903239

[CIT0061] Vittinghoff, E., & McCulloch, C. E. (2007). Relaxing the Rule of Ten Events per Variable in Logistic and Cox Regression. *American Journal of Epidemiology*, *165*(6), 710–718. 10.1093/aje/kwk052 17182981

[CIT0062] Willmot, P., & Mason, A. (2023). Institutional firesetting in a forensic inpatient population. *The Journal of Forensic Psychiatry & Psychology*, *34*(3-4), 386–400. 10.1080/14789949.2023.2240310

[CIT0063] Wilpert, J., van Horn, J., & Eisenberg, M. (2017). Arsonists and violent offenders compared: Two peas in a pod? *International Journal of Offender Therapy and Comparative Criminology*, *61*(12), 1354–1368. 10.1177/0306624X15619165 26721901

[CIT0064] Wyatt, B. (2018). An examination of risk factors and dangerousness associated with firesetters (70875) [MPhil thesis]. University of Kent. Kent Academic Repository. https://kar.kent.ac.uk/70875/

[CIT0065] Wyatt, B., Gannon, T. A., McEwan, T. E., Lockerbie, L., & O’Connor, A. (2019). Mentally disordered firesetters: An examination of risk factors. *Psychiatry*, *82*(1), 27–41. 10.1080/00332747.2018.1534520 30407126

